# The modules of mental health programs implemented in schools in low- and middle-income countries: findings from a systematic literature review

**DOI:** 10.1186/s12889-020-09713-2

**Published:** 2020-10-20

**Authors:** Solomon Musa Gimba, Paul Harris, Amornrat Saito, Hyacinth Udah, Averil Martin, Amanda J. Wheeler

**Affiliations:** 1 Menzies Health Institute Queensland, Griffith University, Brisbane, Queensland Australia; 2 Department of Nursing Science, University of Jos, Jos, Nigeria; 3 School of Nursing and Midwifery, Griffith University, Brisbane, Queensland Australia; 4 Social Work and Human Services, James Cook University, Townsville, Queensland Australia; 5 Academic Engagement Services, Griffith University, Brisbane, Queensland Australia; 6 Faculty of Medical and Health Sciences, University of Auckland, Auckland, New Zealand

**Keywords:** Secondary school, Mental health programs, Adolescents, LMIC

## Abstract

**Background:**

Secondary schools in low- and middle-income countries (LMICs) provide health promotion, preventive, and early intervention services. Nevertheless, literature indicates that the modules of these services are either adapted or modified from existing mental health programs in developed countries. The literature also highlights the provision of non-comprehensive services (mental health promotion, prevention, and early intervention), in LMICs. These findings inform the need for undertaking this systematic literature review. The aim of this review was thus to identify the modules of school-based mental health programs (SBMHP) that have been implemented in LMICs to guide the development of a culturally sensitive comprehensive mental health program for adolescents in a LMIC country.

**Methods:**

The Preferred Reporting Items for Systematic reviews and Meta-Analyses (PRISMA) statement was used to guide this review. The following databases were searched in September 2018, to identify the relevant literature: PubMed, CINAHL, Scopus, Web of Science, PsycINFO, and ERIC. The search was conducted by the first author and reviewed by the authors.

**Results:**

Following the screening process, a total of 11 papers were identified and reviewed for quality. The systematic review highlighted that the mental health programs provided in schools included: an introduction module, a communication and relationship module, a psychoeducation module, a cognitive skills module, a behavioral skills module, establishing social networks for recovery and help seeking behavioral activities and a summary/conclusion module.

**Conclusion:**

This review sheds light on the characteristics of the programs in LMICs. Two programs were found to be universal in nature. Five programs were directed at key risk factors or at-risk groups, and four were early intervention programs. The review also revealed that only one program out of the 11 programs included modules for parents. The synthesis indicated that all the identified programs were adapted or modified from existing programs. The dearth of comprehensive programs in LMICs was also revealed. Lastly, the review revealed seven modules that can be useful for developing a SBMHP.

**Supplementary information:**

**Supplementary information** accompanies this paper at 10.1186/s12889-020-09713-2.

## Background

The provision of child and adolescent mental health (CAMH) interventions in schools has gradually taken centre stage in the global discourse [1–4]. Available literature highlights that schools play a major role in the provision of, and improving, access to mental health interventions to children and adolescents [2, 5–13]. Evidence from high-income countries (HICs) indicates that several programs have been developed and implemented to meet the mental health needs of children and adolescents [14–20]. While this is the case in HICs, little is known about the development of these programs in low- and middle-income countries (LMICs). The available literature in LMICs reveals that programs that have been implemented are either adapted and/or modified from HICs [21–29].

The potential benefits of mental health programs implemented in schools have also been highlighted in LMICs. It is increasingly recognized that universal mental health services provided in schools and other community settings, such as workplaces, are more acceptable than non-community settings because they limit stigmatization and discrimination [30, 31]. Other scholars [31, 32] have also demonstrated that community mental health services are reducing stigmatization and discrimination through mental health promotion, prevention, and intervention in respect of mental health disorders. Indeed, the gap between the burden of mental illness and access to mental health services in LMICs can, in part, be addressed by investing in school-based mental health programs (SBMHP) and other community mental health services [33]. The research suggests that mental health services provided in the school settings have far-reaching benefits for students and for increasing access to services.

The economic benefits of providing mental health services in schools have also been reported in the literature. The return on investment of early identification and intervention programs, such as SBMHP, has also been recognized [34, 35]. These include reducing crime, raising earnings, and promoting education [34, 35]. For instance, early mental health interventions, especially during adolescence, have been associated with prevention of lifetime disability for most people with mental health disorders [35]. Prevention of diseases and health promotion was also identified by the authors as potential distal economic benefits of early life interventions [34, 35]. It appears that, by investing in SBMHP, access to CAMH interventions can be improved in a way that is effective and valued by students in the short term, while realizing distal economic benefits.

Hence, experts are advocating for comprehensive mental health services within school environments and other community settings, such as workplaces and homes [36, 37]. For instance, the mental health promotion interventions continuum (MHPIC) is a group of primary and secondary prevention strategies used in a school community to provide a range of mental health services or interventions [1, 36, 37]. The three levels of the MHPIC are commonly referred to as universal, selective, and indicated [36]. When these three levels are provided in a school or community setting, they are referred to as a comprehensive mental health program [38]. The universal approach focuses on providing interventions across the school population, i.e., all students [39]. The main aim of these programs is to make the school environment free of mental health stressors or predisposing factors by offering access to the programs to students, teachers, and the school community [29]. Reduction of stigmatization is one of the most important impacts of such a universal approach [36]. Selective approaches, in contrast, target groups of students and sometimes their family members who are susceptible to presenting with mental health problems [40]. These programs are mostly preventive [41] and are administered primarily to prevent the development of mental health problems [36]. The main effects of these programs include reduction of disruptive behaviors, depressive symptoms, and the promotion of feelings of togetherness. These programs further provide parents with mental health knowledge and skills that affect their responses to their children’s behavior [36]. The indicated approach focuses on individual students and their family members who have manifested early signs and symptoms of mental health problems [37]. The goal of these interventions is thus the early identification and intervention of mental health problems to prevent or reduce the severity of these and the further development of symptoms. These programs furthermore help to reduce school disciplinary actions, depressive symptoms, and referrals to specialist mental health services [36]. The MHPIC approaches proffer different solutions to different populations within the school community. This indicates that implementing a comprehensive mental health program will allow for wider coverage and multiplier effects in terms of population and solutions, respectively.

The available literature on the provision of culturally responsive comprehensive CAMH in LMICs is scarce. The available literature indicates that the majority of the ongoing child and adolescent research in LMICs has been aimed at identifying the burden of emotional, cognitive, and behavioral problems; needs related to resources; and the availability of resources for developing and implementing mental health programs in schools [6, 9, 42–45]. The scarcity of literature in this field supports the need for further studies that focus on developing culturally responsive mental health intervention programs. The only literature that describes a mental health program for adolescents suggests an existing indicated program in a HIC was adapted for an LMIC [46]. This clearly reveals that there is no existing LMIC literature that describes a culturally responsive comprehensive mental health program. Such a dearth of published studies on SBMHP underscores the need for further research about SBMHP in LMICs in general.

Thus, this systematic literature review sought to synthesize the literature regarding mental health programs in schools, with a view to identifying the modules of the SBMHP that have commonly been implemented in LMICs. The identified modules were used to guide the data collection process and the development of a culturally responsive comprehensive mental health program for schools in a LMIC. The modules were also identified to promote the use of effective modules as baseline for the development of future programs. It is our belief that mental health programs implemented in schools in LMICs may be more beneficial than programs implemented in other community settings and mental health institutions.

## Methods

The programs implemented in LMICs are either adapted from existing programs in HICs or focused on specific mental health problems [21–29, 46, 47]. The need to identify modules from the literature to guide the development of a culturally sensitive program for LMIC was considered imperative. Thus, the current review looked at programs that had been developed and implemented in LMICs and identified modules of mental health programs based in schools.

### Search strategy and eligibility criteria

**Fig. 1:**
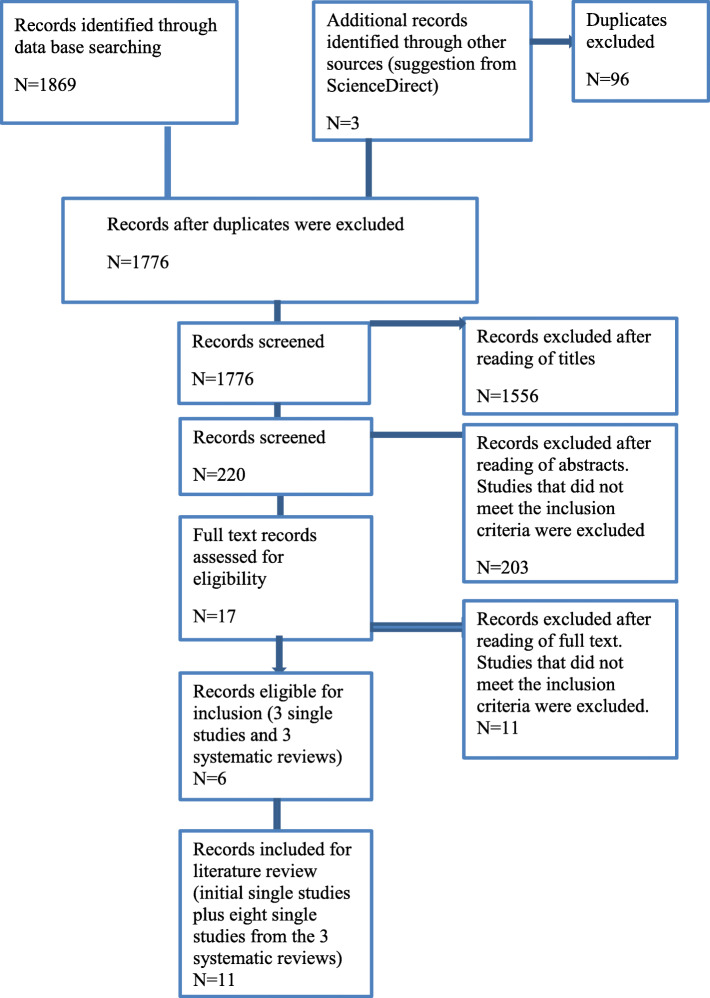
PRISMA flow diagram (2003-2018)

The Preferred Reporting Items for Systematic reviews and Meta-analyses (PRISMA) statement [48] was used to select and refine all possible studies for inclusion in the study. Each step of the literature review was conducted according to the PRISMA statement (see Fig. 1). Articles were selected for inclusion based on the following selection criteria: the study must have been conducted in a school environment; it must have been undertaken with adolescents (12–18 years); and it must have described the modules of the mental health programs. This study focused specifically on adolescent populations of secondary schools; therefore, the exclusion criteria were studies conducted with a combination of children and adolescents, and studies undertaken in HICs.

The search was conducted in September 2018 by the first author. The following databases were searched: PubMed, Web of Science, Scopus, CINAHL, ERIC, and PschINFO. The reference list of full text articles, especially systematic literature reviews, was also searched for articles that met the inclusion criteria [49]. The limiters used were year of publication (2003–2018), peer review, English, human(s), and full text.

The search terms used were mental health, or psychological health, or psychological wellbeing, or life skills, or empowerment, or resilience, or social emotional, or mental health literacy, or mindfulness, AND secondary school, or high school, or junior high school, or middle school, or grades 7–12, AND programs*, or therapy, or intervention, or education, or training, or promotion, or prevention. A summary of the number of articles retrieved is presented in Table 1 (Figure Legends)**.**

**Table 1:** Summary of the Number of Articles Retrieved

S/No	Database	Results (collected between 2003 and 2018)
1	ERIC^a^	740
2	PubMed^a^	19
3	Web of Science^b^	455
4	CINAHL^a^	217
5	Scopus^c^	5
6	PsychINFO^a^	436
**Total**		***N*** **= 1872**

Key: ^a^advanced search, ^b^basic search & ^c^document search

A total of 1872 articles were generated, and all were screened against pre-specified inclusion criteria. A total of 96 duplicates were excluded, resulting in 1776 unique articles for screening. The titles of the 1776 articles were read by three of the authors, and 1556 articles were identified as falling outside the scope of the review. The abstracts of the remaining 220 articles were all read by three of the authors, and thereafter a total of 203 were excluded for not meeting the inclusion criteria. The full texts of the remaining 17 articles were read, resulting in a further 11 articles being screened out, and six full-text articles were read again by the same authors. Of these six articles, three were single studies, while the other three were systematic reviews. The three authors then re-read the three systematic reviews, and eight articles mentioned in these three systematic reviews met the inclusion criteria. Therefore, the eight articles from the systematic reviews and the three single studies were included in this review; giving a total number of 11 articles (see Fig. 1).

### Methodological quality assessment

The Grading of Recommendations Assessment, Development and Evaluation (GRADE) system for rating the quality of evidence and strength of recommendations [50] was used to assess the quality of the 11 studies. The quality of evidence assessed the study design, the quality of the study and its consistency [51].

The GRADE system also highlighted the fact that studies are classified into observational and randomized trials [51]. In scoring a randomized control trial (RCT), high-quality evidence is awarded the maximum score (4 points), but factors such as study limitations, inconsistency of results, indirectness of evidence, imprecision, and reporting bias can influence the confidence in the evidence, thereby reducing the score to moderate (3 points) or low (2 points) [45]. Conversely, the scoring of observational studies starts from low quality (2 points) and may be upgraded to moderate quality (3 points) if the magnitude of the intervention is large [50].

In addition, when further research is not likely to influence the confidence in the estimate of effect of an RCT, the evidence is said to be of high quality (4 points). Evidence is said to be of moderate quality if further research is likely to have an important impact on the confidence in the estimate of effect, and it may change the estimate. Furthermore, evidence is considered low quality if further research is likely to change the findings, and very low quality when the results appear to be very uncertain [50, 51].

## Results

### Characteristics of the programs

As shown in Table S2, all the studies included were from middle-income countries (MICs); seven were from upper middle-income countries [21–27] and four were from lower middle income countries [28–30, 47], as indicated by the World Bank [52]. Three studies were conducted in South Africa [21, 22, 24], two in Bosnia and Herzegovina [26] and one study each was from India, Kosovo, Nigeria, Mauritius, Thailand, and Palestine [23, 27–30, 47]; Africa accounted for five studies (three from South Africa and one each from Nigeria and Mauritius).

A range of experimental designs was employed across the chosen studies, including quasi-experimental [21, 24, 27], Solomon four group design [22], experimental design (RCTs) [23, 26, 28, 30], mixed study design [24], intervention study [47] and a cross-sectional cohort study [29]. Sample sizes differed significantly: the smallest sample was 12 [24], while the largest was 877 [29]. The quality of the studies also differed based on the GRADE system assessments: two studies were of high quality [23, 26], seven were moderate [21, 26–29, 47], and two were low quality [22, 24]. This suggests that most of the studies had adequate quality ratings.

Practical indices, such as the duration of the programs and who conducted the programs, were also evaluated. The duration of individual sessions of the programs ranged from 45 min to 12 h. The number of weekly sessions per programs ranged from one to three sessions per week. The total duration for implementing the individual programs ranged from 3 weeks to 1 year [21–30, 47]. The programs were implemented by a range of professionals, including teachers [21, 23, 27, 30], school counsellors [26, 29], researchers and research assistants [28], consultant psychiatrists, [47] and psychologists [22]. This highlights the culture of the multidisciplinary approach in the provision of mental health interventions in schools.

The involvement of stakeholders in the development of the programs was also highlighted. Out of the 11 programs, one program was developed through needs assessments conducted with multiple stakeholders, including students, parents, non-governmental organizations (NGOs), and policy makers [27]. Others were developed by the researchers [28] or adapted from existing programs [47], while in some others, this was not indicated [21–23, 26, 28–30].

The effectiveness of the 11 programs varied in relation to the individual outcomes of the programs. Five programs [23, 26, 29, 30, 47] were significantly effective across all measured outcomes, and were measured after a period that ranged from 3 months to 4 years. The effects of the five programs on adolescent mental health were maintained throughout the measured periods. One [23] of the programs, however, revealed different effects due to the maintenance dose. Improvements in self-esteem and coping skills were maintained at 6 months’ follow-up, while improvements in depression symptoms and hopelessness were not maintained at 6 months’ follow-up [23]. Although three of the programs indicated improvements across all the outcomes [24, 26, 28], but they did not measure the effects after the implementation.

The remaining three programs [21, 22, 27] showed varying effects. One of the articles revealed that there was a significant improvement in interpersonal strength, emotional regulation, self-appraisal, and emotional reactivity, and these were also maintained at 3 months’ follow-up [22]. Also, no significant improvement was reported in family involvement, intrapersonal strength, school functioning, affective strength, sense of mastery, sense of relatedness, family appraisal, or general social support [22]. Another study [21] indicated significant increase in intrinsic motivation, decreased introjected motivation and amotivation in the intervention group. For the control group, there was a sharp increase in recent and heavy use of alcohol and cigarettes. The effects of the programs on alcohol and cigarette use were found to be greater for girls [21]. Significant improvement in self-esteem, perceived self-efficacy, pro-social behavior, and perceived adequate coping was reported. Participants showed significantly better adjustment in respect of teachers, better adjustment in school, and improved classroom behavior. However, no change was observed in adjustment in respect of parents and peers [27].

### Description of the program modules

#### Modules of the universal programs

Universal programs were identified in two of the studies [26, 27]. The modules of these programs included psychoeducation, relationship and communication, cognition, and coping skills modules. The psychoeducation module covered topics such as introduction of participants and areas to be covered in the programs, self-introductions, and building rapport. The second module dealt with relationships and communication, and it covered self-awareness, empathy, learning how to be friendly, and learning how to communicate with friends. The cognition module, which was the third module, covered topics such as problem-solving skills and anger management, decision-making, and critical and creative thinking. The final module was related to coping skills; for example, how to manage emotion and stressful situations. Both programs targeted all the school students and/or parents, but not the teachers [26, 27].

#### Modules of the selective programs

A total of five programs were selective in nature [21–23, 26, 29]. The modules of the selective programs were described based on the target population. The target population categories included: 1) children predisposed to or experiencing mild cognitive, emotional, and behavioral problems; 2) children at risk for sexual behavior and substance abuse; 3) children who were victims of war; and 4) children living in conflict-prone areas.

##### Mild cognitive, emotional, and behavioral problems

The modules of the program targeted children predisposed to or experiencing mild cognitive, emotional, and behavioral problems. The program included the introduction, relationship and communication, behavioral and cognitive modules for students and the behavioral module for teachers. The introduction module introduced participants to the areas to be covered in the programs [22]. The second module, viz., the relationship (intra- and interpersonal relationship) and communication skills, included developing a strong sense of identity, developing and maintaining realistic self-esteem, identification of emotions, expression of emotions and basic communication skills. Cognition, the third module, covered topics like conflict management, assertiveness, and tolerance regarding diversity [22]. Behavioral skill was included in the fourth module, and it dealt with teaching students successful time management and adaptability [22].

##### Sexual behavior and substance abuse

The program modules included drug-related psychoeducation and sexual relationship and cognition modules [22]. Drug-related psychoeducation covered topics around the definition of drugs, signs and symptoms. The relationship module, the second module, covered topics such as self-awareness and leisure activities. The third module was cognitive skills, which included problem-solving activities, decision-making activities, and coping skills activities [21].

##### Victims of war

The programs targeting children who were victims of war included modules on relationship and communication, trauma related psychoeducation and training topics, cognitive, social support for recovery, and behavior. The first module covered topics like self-awareness and self-esteem activities, building trust and sharing concerns [26, 29]. The second module was trauma-related psycho-education and training, which covered the following topics: learning about emotions, how to control emotions via bodily and verbal processes and regulating breathing, and somatic problems [26, 29]. The cognitive module was third and included problem identification and problem-solving skills. Examples of problem identification skills included writing about and drawing traumatic events (frightening, disturbing experiences; dreams or memories). Problem-solving skills, such as talking about traumatic events to third parties, storytelling, and exploration of emotions were also included. Other activities included coping skills, relaxation and breathing exercises, sleep, and role playing [27, 29]. The fourth and fifth modules covered topics such as help-seeking behavior and recovery process activities [27, 29].

##### Conflict-prone areas

The programs that targeted children living in conflict-prone areas covered topics related to students and their parents. The modules for children included psycho-educational topics and relationship-building activities, cognition, and social networks. The psycho-educational topics and relationship-building activities related to family harmony and avoiding the escalation of conflicts [23]. The third module covered cognition-related topics and problem-solving skills (stress inoculation techniques, trauma processing through narrative drawings, and reactions during and after times of danger) [23]. Establishing social networks was part of the fourth module [23].

This program also included activities for parents. Session one involved identification of existing parental strengths and stressors, followed by management of stress to enhance calm and effective parenting; session two offered information about normal adolescent development and strategies for promoting self-esteem and balancing independence and attachment issues; and session three provided strategies to promote family harmony and manage conflicts [23]. The modules covered by all five selective programs included introduction, psychoeducation, relationships and communication, cognition, behavior, and social support systems. These modules resembled those of the indicated programs (see below).

#### Modules of the indicated programs

Four programs [24, 28, 30, 47] were indicated, which targeted adolescents with depression, learning disabilities, and negative thinking. The modules covered in these programs included an introduction, psychoeducation, intra-communication, and relationships, cognition, and a conclusion. The first module focused on introductory activities, such as exchanging pleasantries [28, 30, 47]; the second focused on psychoeducation, such as signs and symptoms of depression [47]; the third on intra-communication and relationship activities, such as stabilization, self-actualization, and self-esteem-related activities [24, 28]. The fourth module covered cognitive activities, for example, identification and listing of daily pleasurable activities, identification of emotions, controlling emotions via coping skills, relaxation activities, and problem-solving activities such as boosting self-esteem, storytelling trauma narrative activities, and resilience activities [24, 28, 47]. The conclusion, summary and revision made up the fifth module [24, 28, 47].

The systematic review highlighted that the mental health programs provided in schools were made up of the following modules: an introduction module, a communication and relationship module, a psychoeducation module, a cognitive skills module, a behavioral skills module, establishing social networks for recovery and help seeking behavioral activities module and a summary/conclusion module.

## Discussion

The current systematic review was undertaken to identify the modules of mental health programs implemented in schools that could be used to develop a culturally responsive comprehensive mental health program to be implemented in schools for adolescents (12–18 years) in LMICs. To the best of our knowledge, this is the first systematic review to be conducted within the LMIC literature, primarily to identify possible effective modules of mental health programs that can be implemented in schools for adolescents [52–59].

Our review, although it is the first to be undertaken in LMICs, is the second to be undertaken globally. A study conducted by Skeen et al. [60], is the first study that was aimed at identifying the modules of mental health programs implemented in schools. The findings of our review and those of Skeen and co-authors [60] share some similarities and dissimilarities. The quality of the body of evidence of the studies included in our review was assessed using GRADE. The studies included in the first study [60] did not use GRADE or any assessment tool. According to Skeen et al. [60], the studies included in their review were not assessed for quality. This could potentially influence the bias in relation to the quality of the studies included in both reviews. There are also some similarities with both studies in terms of their limitations. In this review, one of the studies did not indicate if there was allocation concealment or random sampling. In the first review [60], allocation concealment and random sampling were also not done in some studies. In relation to the research designs employed, the current review included studies that utilised both real life setting designs and research setting designs (i.e., RCTs and quasi-experimental designs). In the review undertaken by Skeen et al. [60], the studies reviewed utilised only experimental designs. The implication of this is that, while the findings of our review can be applied in both research settings and non-research settings, the findings from the first review undertaken [60], may only apply to research settings.

The review conducted in the current study confirmed the claim by [53] that there is a dearth of literature on SBMHP for adolescents in LMICs. This finding is in line with other reviews undertaken in LMICs, which has been attributed to a dearth of professionals, acceptability of interventions [60, 61], poor funding of mental health by LMICs and a shortage of open access publications [60, 62, 63]. The finding of this current study agrees with the finding of [52] which supports the need for developing culturally responsive and comprehensive mental health programs for schools in LMICs, advocating for more funding of mental health programs for adolescents by LMICs and undertaking more school-based mental health research by professionals. The fact that the number of SBMHPs was higher in Africa than in any other region [52] implies that African countries are increasingly becoming responsive to the global discussion about mental health promotion and prevention in schools.

The current review indicated that the effectiveness of the 11 programs varied in relation to the individual outcomes of the programs and the period of follow-up. This finding agrees with that of another study, which revealed that programs implemented by teachers were more effective than those implemented by other stakeholders, such as psychiatrists and researchers [64]. This implies that program development should be outcome-dependent and that it should be followed up effectively and efficiently.

Regarding effectiveness, all the programs were effective. This finding is consistent with other existing literature. For instance, Lyn and co-authors [52] reports that SBMHP implemented in LMICs have significant positive effects on students’ emotional and behavioral wellbeing, including reduced depression and anxiety and improved coping skills.

Furthermore, one of the studies included in the review had modules for both parents and adolescents. Feedback from the parents recruited into the study revealed that the parent module allowed for improvement in the compliance of the adolescents to the intervention regimen, which in turn, positively affected the outcomes. This supports the finding that programs that target multiple stakeholders may be more effective [2].

In our systematic review, five of the 11 programs were identified as selective and four as indicated, while two studies were universal. This highlighted that the universal programs were notably fewer. This is in contrast with the report of another study conducted in Australia (a HIC), which revealed that the universal program accounted for more than half of the programs included in the review [62].

Our systematic review indicated that seven modules were included in the 11 studies: an introduction, psychoeducation, relationship and communication, cognition, social support systems, behavioral and conclusion modules. These indicate the range of modules that have commonly been utilized in LMICs, and that hence can also be used to guide the development of future mental health programs to be implemented in schools in LMICs. Some of the modules identified in this review reflect those reported in the review by Skeen et al. conducted in 2019 [60]. For instance, interpersonal relationship and emotional stability were highlighted as modules in the 2019 study. These modules are similar to the communication and relationship module found in this review. Conversely, the other modules, which constitute most of the modules, differ in both studies. This could indicate that different settings in terms of geography may influence the applicability of a module or modules.

### Study limitations

This systematic review has a few important limitations. The first is related to the scope of the systematic search. Due to the time scale and resources available, a systematic search for studies published in the grey literature (i.e., research and materials that are unpublished or that have been published by individuals and organizations outside the traditional commercial or academic environment) was not included. Furthermore, the search did not consider languages other than English and, therefore, studies in the other former colonial languages of French, Spanish, Portuguese, and Dutch were not included. The second set of limitations related to the selection criteria. The studies included were all peer reviewed, hence there is possibility that some programs were not identified. Another important limitation of the study is the fact that our search strategy missed eight relevant articles that were only found through other systematic reviews. This suggests that our search strategy/search terms were not comprehensive enough.

## Conclusion

The systematic literature review indicated the unavailability of universal and comprehensive programs in LMICs. It showed that two programs were universal programs, and that no comprehensive programs were available, thus highlighting the need to develop comprehensive SBMHP in LMIC settings. Furthermore, the systematic literature review revealed that one of the programs incorporated modules for parents [29]. This finding indicated the need to develop a culturally sensitive, comprehensive SBMHP that incorporates modules for adolescents, their parents, and their teachers.

The literature review also revealed seven major program modules, which include an introduction module, a communication and relationship module, a psychoeducation module, a cognitive skills module, a behavioral skills module, a module on establishing social networks for recovery and help seeking behavioral activities, and a conclusion module. These options will form the basis for further research, consultations, and the development of a SBMHP in an LMIC.

## Supplementary information

**Additional file 1: Table S2.** Systematic Literature review of SBMHP for adolescents in LMIC.

## Data Availability

The data and materials used in this study can be access through the databases used. See supplementary table 2.

## Abbreviations

CAMH: Child and adolescent mental health
GRADE: Grading of Recommendations Assessment, Development and Evaluation
HICs: High Income Countries
LMICs: Low and middle-income countries
MHPIC: Mental health promotion interventions continuum
MICs: Middle-income countries
NGO: Non-governmental organization
RCT: Randomized controlled trial
SBMHPs: School based mental health programs
