# Supplementary material for: The modules of mental health programs implemented in schools in low- and middle-income countries: findings from a systematic literature review
Source: BMC Public Health. 2020 Oct 20;20:1581. doi: 10.1186/s12889-020-09713-2 (PMC7576731; doi:10.1186/s12889-020-09713-2)
Supplement: Supplementary file 1 — Additional file 1: Table S2. Systematic Literature review of SBMHP for adolescents in LMIC. [file 12889_2020_9713_MOESM1_ESM.docx]

**Table 2: Systematic Literature review of SBMHPs for adolescents in LMIC**

| **S/No** | **Author/year/country** | **Target population** | **Name of program** | **Type of programme** | **Components of programme** | **Effectiveness/Quality of evidence** |
| --- | --- | --- | --- | --- | --- | --- |
| 1 | Layne et al (2008)  Bosnia & Herzegovina | 159 students were selected from ten high schools in central Bosnia | classroom-based psychoeducation and skills intervention with or  without an additional trauma and grief component | Universal | Psychoeducation and learning skills intervention. Psychoeducation about reactions to trauma; enhanced coping; trauma and grief processing; building of social support skills; enhanced problem solving; understanding of links between behaviour and trauma; and reappraisal of traumatic expectations. | - Significantly effective in PTSD, depression, existential and traumatic grief. The improvement of the outcomes was maintained after four months - High quality |
| 2 | Cox et al., (2007)  Bosnia & Herzegovina | 34 students from 10 secondary schools throughout central Bosnia | A school-based trauma/grief focus group treatment programme | Selective | Trauma related psychoeducation; learning and/or practicing one or more coping skills; processing therapeutically issues related to trauma exposure; loss; post war adversities; or maintaining healthy adolescent development; and processing students here and now interactions in the group. | - Significantly effective - Moderate quality |
| 3 | Bella-Awusah et al (2016)  Nigeria | 40 secondary school students (ages 14–17) who were diagnosed of depression in Ibadan, South West, Nigeria | School based cognitive behavioural therapy | Indicated | Psychoeducation on causes; symptoms and treatment of depression; cognitive techniques for generating; and using positive self-talk; religious based coping self-talk, and the link between cognition; emotion and behaviour; Psychoeducation on rational and behavioural activation. The students were taught how to identify pleasurable activities and activities to avoid as well as monitoring of their mood. Psychoeducation on identification of pleasurable activities; and listing of daily pleasurable activities. Relaxation techniques like deep slow breathing exercises; and positive imaging. Revision of the proceeding sessions and techniques. | - Significantly effective for improving depressive symptoms and knowledge base on depression. The improvement of the depressive symptoms was maintained after 16 weeks post treatment with an additional improvement in psychological functioning - Moderate quality |
| 4 | Rivet-Duval et al., (2010)  Mauritius | 160 (12-16 years) adolescents were randomly selected from two public schools in Mauritius. | School-based intervention designed for conflict exposed children | Selective | Components for adolescents (RAP-A)  Establishing rapport; Affirmation of existing strengths; promoting self-management and self-calming skills in the face of stress; cognitive restructuring; problem solving; building and accepting psychological support networks; interpersonal components designed to promote family harmony; and avoid escalation of conflict; summary and termination  Components for family (RAP-F)  Identification of existing parental strengths; and identification and management of stress to enhance calm; and effective parenting; information on normal adolescent development and strategies for promoting self-esteem and balancing independence and attachment issues; provision of strategies to promote family harmony and manage conflicts | - Significant improvement in depressive symptoms, hopelessness, self-esteem, coping skills. Improvements in self-esteem and coping skills maintained at 6 months follow up. Improvements in depression symptoms and hopelessness not maintained at 6 months follow up - High quality |
| 5 | Srikala et al., (2010)  India | 605 adolescents of both sexes ages 14 to 16 years studying in 8th, 9th or 10th standard were selected from two schools in (Bangalore rural and Udupi) | Life skills education programme (LSE program) -NIMHANS model | Universal | Critical thinking and creative thinking; decision making and problem solving; communication skills and interpersonal relations; coping with emotions and stress; and self-awareness and empathy | - Significant improvement in self-esteem, perceived self-efficacy, pro-social behaviour and perceived adequate coping. Participants showed significant better adjustment with teachers, better adjustment in school and improved classroom behaviour. No change was observed in adjustment with parents and peers - Moderate quality |
|  |  |  |  |  |  |  |
| 6 | Caldwell (2004)  South Africa | 345 secondary school students grade 8–9 (mean of 14 years) in low income township in Cape Town were selected (Targeted risk behaviours included substance use and sexual risk behaviour) | HealthWise South Africa | Selective | Grade 8  Self-awareness; managing anxiety; managing anger; exploring free time; free time in my community; beating boredom and developing interest; overcoming roadblocks; decision making; managing risk; avoiding risky sexual behaviour; myths & realities of drug use; and avoiding and reducing risk  Grade 9  Review; leisure motivation; community connections; planning and managing leisure; relationships and sexual behaviour; conflict resolution | - Significant increase in intrinsic motivation, decrease in introjected motivation and amotivation, increase in perception of condom availability in intervention group. Control group had ‘steeper increase’ in recent and heavy use of alcohol and cigarette use. Programme effects on alcohol, cigarette use greater for girls. - Moderate quality |
|  |  |  |  |  |  |  |
| 7 | De Villiers et al., (2012)  South Africa | 161 twelve-year-old students were selected from four schools in the Bloemfontein area exposed to numerous family and economic risks as well as adverse life events | Resiliency programme for children | Selective | Introduction of the participants, the programme and the explanation of ethical rules; intrapersonal skills -having a strong sense of identity and how to develop and maintain realistic self-esteem; intrapersonal skills - identifying, expression and management of emotions. Participants practised skills to enable them to identify emotions in themselves and others; interpersonal skills - basic communication skills, conflict management and assertiveness; interpersonal skill -tolerance about diversity; cognitive skills -problem solving was presented in two sessions; cognitive skills - goal setting and motivation; and behavioural skills - successful time management and adaptability provide an important resource with which to manage the environment. | - Significant improvement in interpersonal strength, emotional regulation, self-appraisal, emotional reactivity. Improved self-appraisal scores maintained at three months follow up. No significant improvement in family involvement, intrapersonal strength, school functioning, affective strength, sense of mastery, sense of relatedness, family appraisal, general social support. - Low quality |
| 8 | Theron (2006)  South Africa | 12 adolescents from a special secondary school in South Africa | School-based resilience programme | Indicated | Introductory session: the need for resilience; self-knowledge; internal locus of control and choices; attitude and anxiety; assertiveness skills; faulty thinking; personal bill of rights; empowerment; future orientation and drive; social orientation; self-concept; and closure | - Significant improvement in positive future orientation. Improvement in positive attitude, assertiveness, enthusiasm, drive, anxiety and good interpersonal relationship. The improvement in positive self-concept and internal locus of control was marginal. The improvement of the outcomes was not evaluated. - Low quality |
| 9 | Kaesornsamut et al (2012)  Thailand | 60 Thai high school students selected from a public school in Bangkok | BAND intervention | Indicated | Introducing the program, developing rapport and trust, Learning basic skills for developing relationships; building a sense of belonging, diminishing the feeling of being an outsider; engaging in two-way communication; Using constructive and destructive communication; identifying the characteristics of negative thinking; identifying stressful situations and their related negative emotions and behaviours; learning relaxation, techniques, learning effective problem-solving strategies; and reflecting on what was learned and how to apply in one’s life, expressing, constructively, feelings and behaviours. | - Significant improvement in sense of belonging, negative thinking and depressive symptoms. The maintenance of the improvement of the outcomes was not measured up - Moderate quality |
| 10 | Constandinides et al 2011  Palestine | 877 adolescents (476 boys and 401 girls) between  the ages of 13 and 15 years were selected from the seventh, eighth, and ninth  grades of 23 schools within Qabatia district | School based psychosocial programme (SBPSP) | Selective | Introductions; group building; ice-breaker activity; group story, being a good leader (finding the leader within oneself); my feelings, identifying and coping with emotions in a nonthreatening atmosphere; coping with fear finding positive ways to deal with fear, physical expression and coping with scary sounds; transforming a specific event into a story,  stimulating imagination, cooperation and expressive skills; from a story into a picture, creating pictures for stories; dealing with current and accumulated stress; self-awareness; how do I behave, looking for creative ways to solve conflicts; and closing; wrap-up of the sessions, good-bye celebration | - Significant improvement in psychological wellbeing. It was maintained for at least four years - Moderate quality |
| 11 | Gordon et al., (2008)  Kosovo | 82 students aged 14- 18 were selected from a high school in Suhareka | Mind body skills | Indicated | Guided imagery; relaxation techniques; active techniques to reduce stress; self-expression through words, drawing and movements; and genograms to explore the emotional support in the families | Significant improvement in PSTD symptoms, which was maintained at the 3 months follow up/Moderate quality |
